# The Relationship Between Food Craving, Appetite-Related Hormones and Clinical Parameters in Bipolar Disorder

**DOI:** 10.3390/nu13010076

**Published:** 2020-12-29

**Authors:** Martina Platzer, Frederike T. Fellendorf, Susanne A. Bengesser, Armin Birner, Nina Dalkner, Carlo Hamm, Melanie Lenger, Alexander Maget, René Pilz, Robert Queissner, Bernd Reininghaus, Alexandra Reiter, Harald Mangge, Sieglinde Zelzer, Hans-Peter Kapfhammer, Eva Z. Reininghaus

**Affiliations:** 1 Department of Psychiatry and Psychotherapeutic Medicine, Medical University of Graz, 8036 Graz, Austria; martina.platzer@medunigraz.at (M.P.); susanne.bengesser@medunigraz.at (S.A.B.); armin.birner@medunigraz.at (A.B.); nina.dalkner@medunigraz.at (N.D.); carlo.hamm@stud.medunigraz.at (C.H.); melanie.lenger@medunigraz.at (M.L.); alexander.maget@medunigraz.at (A.M.); rene.pilz@medunigraz.at (R.P.); robert.queissner@medunigraz.at (R.Q.); berndreininghaus@web.de (B.R.); alexandra.reiter@stud.medunigraz.at (A.R.); hans-peter.kapfhammer@medunigraz.at (H.-P.K.); eva.reininghaus@medunigraz.at (E.Z.R.); 2 Research Unit on Lifestyle and Inflammation-Associated Risk Biomarkers, Clinical Institute of Medical and Chemical Laboratory Diagnostics, Medical University of Graz, 8036 Graz, Austria; harald.mangge@medunigraz.at (H.M.); sieglinde.zelzer@klinikum-graz.at (S.Z.)

**Keywords:** food craving, ghrelin, leptin, bipolar disorder

## Abstract

Obesity and weight gain in bipolar disorder (BD) have multifactorial underlying causes such as medication side effects, atypical depressive symptomatology, genetic variants, and disturbances in the neuro-endocrinal system. Therefore, we aim to explore the associations between food craving (FC), clinical parameters, psychotropic medication, and appetite-related hormones. In this cross-sectional investigation, 139 individuals with BD and 93 healthy controls (HC) completed the food craving inventory (FCI). In addition, blood samples (including leptin and acylated ghrelin) were analyzed and sociodemographic and anthropometric data were collected. Individuals with BD reported higher frequencies of total FC as well as craving for fat and fast food than HC. Additionally, we found a significant negative correlation between FC and ghrelin levels in BD. Smokers with BD reported significantly more craving for high fat foods than non-smokers. Age was significantly associated with FC independent of group. Individuals with BD taking olanzapine and quetiapine reported higher frequencies of craving for sweet food, while patients currently taking lithium reported less total FC compared to those without lithium therapy. Likewise, patients currently taking valproate reported less total FC and less craving for sweets than those not taking valproate. FC appears to be of clinical relevance in individuals with BD. Contrary to previous data, this does not seem to be a female phenomenon only and might encompass more than the specific craving for carbohydrates. Although due to the cross sectional design, causality cannot be determined, the association between depressive symptomatology and fast food craving warrants further research.

## 1. Introduction

Bipolar disorder (BD) is a severe chronic psychiatric disorder associated with a high risk for obesity and metabolic disturbances [1,2]. This elevated risk heightens the medical morbidity and mortality in this population and is associated with a more severe course of illness and worsened psychiatric outcome [3]. The potential weight-gaining effects of psychotropic medication, disturbances in the neuro-endocrinal system, atypical depressive symptomatology, as well as a genetic predisposition for weight gain have all been proposed as etiogenic factors of obesity in BD [4]. Weight regulation involves complex homeostatic and non- homeostatic mechanisms including central regulatory pathways, peripheral feedback from adipose tissue and the gastrointestinal tract via appetitive peptides as well as psychological factors such as food craving (FC) [5]. FC is defined as “intense desire to eat a certain food or type of food” [6]. It appears to predict weight-related outcomes and might therefore contribute to increasing obesity rates [7]. FC is described as a multidimensional concept, including cognitive (e.g., thoughts about food); emotional (e.g., intense desire to eat); behavioral (e.g., seeking food); and physiological (e.g., salivation) processes [8]. Typically, individuals report craving highly processed, palatable foods [9]. The craving for carbohydrates in particular has been in the focus of previous research. This is due to its association with obesity [7,10], eating disorders [11,12], seasonal affective disorder [13] and the premenstrual syndrome [14]. Additionally, it has been demonstrated that carbohydrate-rich foods like chocolate, other sweets and sweet desserts seem to be the most craved foods in the majority of individuals [15,16,17]. FC seems to be more prevalent in women than in men [16,17,18]. It is common in young adults and evidence suggests that the number of craving episodes decreases as individuals age [19].

Across all research, a strong relation was found between FC and mood. Christensen and Pettijohn [15] demonstrated that in the majority of individuals who were identified as carbohydrate cravers (72% of participants in their study), anxiety, fatigue, and depression preceded craving episodes. In contrast, protein cravers were more likely to report feeling hungry, happy or bored at the onset of craving.

Leptin and ghrelin are the main peripheral inputs that regulate energy homeostasis and the sense of hunger and satiety. They both are linked to food intake and craving. Leptin, an adipokine, is a pro-inflammatory hormone produced primarily by adipocytes. It circulates at levels proportional to body fat and acts as a signal to the brain for the amount of energy stored [20]. Under physiological conditions, leptin limits food intake via stimulation of anorexigenic neurons [21]. In addition, it is involved in immune processes, interacts with the sleep-wake cycle, and regulates sexual behavior along with reproductive function. Previously, increases in leptin levels have been associated with weight gain induced by second-generation antipsychotics (SGA) and lithium [21,22].

Ghrelin is a 28 amino acid orexigenic peptide that stimulates food intake by binding to the growth hormone secretagogue receptor (GHSR) in the hypothalamus and therefore is often considered to be the antagonist of leptin [23]. It is highly conserved across several species and primarily synthesized by gastric neuroendocrine cells [24,25]. Ghrelin is involved in the secretion of growth hormone in the anterior pituitary cells as well as in the central regulation of energy homeostasis [26]. It is relevant for food seeking behavior and meal initiation. Ghrelin is decreased in human obesity; plasma levels are inversely correlated with body mass index (BMI) [27]. Ghrelin levels increase pre-prandially and decrease post-prandially [28,29]. Previous research has suggested that the “biological active” acylated ghrelin may be more responsive to food intake and its motivational effects [25].

Leptin and ghrelin have also been implicated in neuropsychiatric disorders [30,31,32] and might lie at the very crossroads between obesity and mood. Therefore, they are of special interest in regard to FC in affective disorders. The objective of this investigation was to evaluate FC in a cohort of individuals with BD in comparison to healthy controls (HC). We sought to examine potential associations between FC and clinical parameters, medication intake and leptin as well as ghrelin levels. We hypothesized that FC is more common in individuals with BD than in HC and that there is a correlation between FC and the intake of psychotropic medication as well as leptin and ghrelin levels.

## 2. Materials and Methods

This investigation was conducted at the Medical University of Graz, Department of Psychiatry and Psychotherapeutic Medicine as part of the ongoing single center BIPFAT study. The BIPFAT study aims to explore the relationship between BD and obesity, metabolism, lifestyle and cognitive function. For in-depth information about the study design and preliminary results see previously published reports [1,33,34,35,36,37,38,39,40,41,42,43,44]. The study has been approved by the local ethics committee (Medical University of Graz, Austria) in compliance with the current revision of the Declaration of Helsinki, ICH guideline for Good Clinical Practice and current regulations (EK-number: 24-123 ex 11/12).

In this investigation, 139 individuals with a diagnosis of BD type I or BD type II and 93 healthy controls were included. Bipolar participants were recruited at the Department of Psychiatry and Psychotherapeutic Medicine at the Medical University of Graz. To confirm the psychiatric diagnosis, a structured clinical interview [45] was conducted according to the Diagnostic and Statistical Manual of mental disorders (DSM-IV) [46] by either a psychiatrist or a psychologist. Participants had to be of legal age and had to give written informed consent prior to their participation in this study. Participants completed the German version of the Beck Depression Inventory-II (BDI-II) [47]. In addition, the Hamilton Rating Scale for Depression (HAMD) [48], the Young Mania Rating Scale (YMRS) [49], and Global Assessment of Functioning (GAF) were administered.

Complete medical and psychiatric histories, as well as demographic and anthropometric parameters were taken from all participants. In addition, a fasting blood sample was obtained.

Participating individuals completed a German version of the Food-Craving Inventory (FCI) [50], a self-report measure for general and specific cravings. It allows individuals to indicate how often they experienced a craving for a certain type of food over the past four weeks using a 5-point Likert scale (A = Never, B = Rarely, C = Sometimes, D = Often, E = Almost daily). The FCI consists of 28 items that form four factors. These factors correspond to four subscales for specific cravings for foods high in fat, sweets, carbohydrates and starches, and fast food, respectively. The FCI was chosen because it has been evaluated using individuals of both sexes, across relatively diverse ranges of age and BMI [50]. Additionally, it has good test-retest reliability for both the total score and subscales and its criterion validity has been demonstrated previously [51].

Exclusion criteria for participating in the study were the presence of severe medical or neurological comorbidities such as active cancer (including treatment with chemotherapeutic agents), chronic obstructive lung disease, rheumatoid arthritis, systemic lupus erythematosus, Alzheimer’s disease, Parkinson’s disease, Huntington’s disease, or multiple sclerosis. Additional exclusion criteria for HC were a lifetime psychiatric diagnosis and first-degree relation to individuals with psychiatric disorders. No pregnant or breastfeeding female individuals participated in this investigation.

### 2.1. Biological Assays

A blood sample was obtained from all participants between 8:00 and 9:30 in the morning after a fasting period of 12 h. Metabolic parameters were measured immediately. Part of the samples were stored immediately at −80 °C and later thawed for further analyses. Plasma levels of leptin and acylated ghrelin were measured by enzyme-linked immunosorbent assay (ELISA), according to the procedures supplied by the manufacturer (Firma BioVendor, Brno; Czech Republic). The intra- and interassay coefficients of variation for leptin (RD191001100 Human Leptin ELISA) were 7.6% and 4.4%; the limit of detection (LOD) was 0.2 ng/mL. For acylated ghrelin (RA194062400-384R Ghrelin Acylated Human ELISA) the intra- and interassay coefficients of variation were 8.0% and 8.4%; the LOD was 2 pg/mL. Concentrations are expressed as pg/mL (ghrelin) or ng/mL (leptin).

### 2.2. Reference Values

According to Mayo Medical Laboratories [52], reference values for leptin in individuals with a mean BMI of 22 range from 0.7 to 5.3 ng/mL in men and 3.3 to 18.3 ng/mL in women. Reference levels for ghrelin range from 520 to 700 pg/mL in normal weight individuals and from 340 to 450 pg/mL in obese individuals [52]. Levels of acylated ghrelin from 180.4 pg/mL to 411.8 pg/mL have been reported in healthy individuals [53].

### 2.3. Statistical Analyses

Differences in FC and ghrelin and leptin levels were assessed via (M)ANCOVA. *t*-tests or Mann-Whitney-U-tests were used to assess differences in continuous demographic and clinical variables; Chi-square-tests to examine categorical variables. Bivariate and partial correlations were performed to examine associations between parameters. Error probabilities below 0.05 were accepted to denote statistical significance. SPSS version 25 was used to perform data analyses.

## 3. Results

### 3.1. Sample Description

Overall, among the 232 participants, individuals with BD and HC differed significantly in the parameters age, BMI and smoking behavior. Patients were older, had a higher BMI and were more likely to be smokers and male than HC. Within the two groups, male patients and controls had a higher mean BMI than female patients and controls, respectively (refer to Table 1 for details). In the group of male individuals with BD there were 44.6% overweight (defined by a BMI between 25.00 and 29.99 kg/m^2^) and 31.1% obese (defined by a BMI greater than or equal to 30.00 kg/m^2^) participants, respectively. Among female patients, 21.5% were overweight and 30.7% were obese. In the HC group, 40.0% of men and 14.5% of women were overweight, while 17.1% of men and 12.1% of women were obese.

Among patients, 132 were euthymic at the time of study (defined by a HAMD score below 14 and a YMRS score below 9), while seven fulfilled criteria for a mild depressive episode. There were no patients with a (hypo)manic or mixed episode at the time of study.

### 3.2. Food Craving

**Figure 1:**
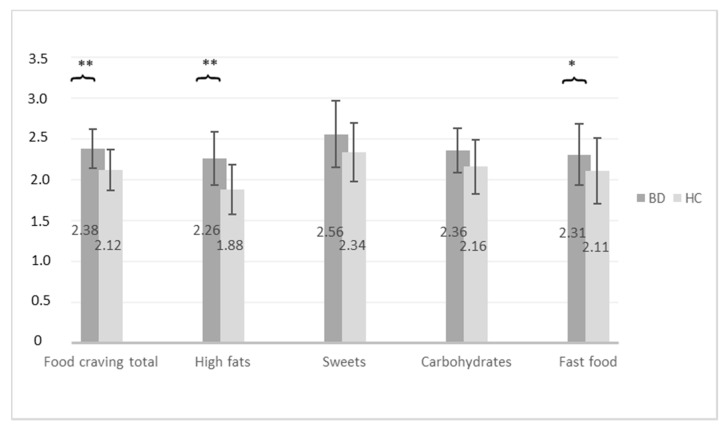
Differences in food craving between individuals with BD and HC (* *p* < 0.05, ** *p* < 0.01). Abbreviations: Bipolar disorder (BD), healthy controls (HC).

Patients reported higher frequencies of total FC (t(230) = 3.83, *p* < 0.001) as well as higher frequencies of three subdomains of FC: fat (t(230) = 4.43, *p* < 0.001), sweets (t(230) = 2.08, *p* = 0.038) and carbohydrates (t(230) = 2.39, *p* = 0.018) (see Figure 1) than controls. After controlling for possible effects of sex, BMI and age, differences in total FC (F(1, 227) = 8.70, *p* = 0.004, partial η² = 0.04), craving for fat (F(1, 227) = 8.86, *p* = 0.003, partial η² = 0.04), and craving for fast food (F(1, 227) = 4.46, *p* = 0.036, partial η² = 0.02) remained significant.

Sweets were the most frequently craved type of food regardless of sex and group. Likewise chocolate was the single food item most frequently craved by patients and controls and men and women alike. Overall, 20.9% of BD participants (16.2% in men; 26.2% in women) and 16.1% of HC (11.4% in men; 19.0% in women) reported almost daily cravings for chocolate. Fried chicken, pork roast, fish fingers, bacon, “Wiener Schnitzel”, cold cuts, wafers, tortes, sponge cake and French fries were significantly more frequently craved by individuals with BD compared to HC (for differences in craving for individual food items refer to Table 2).

No differences between weight groups (normal weight, overweight and obese) in total FC or cravings subgroups were found in individuals with BD. In HC, however, individuals of normal weight reported significantly higher frequencies of total FC than overweight and obese individuals (*F*(1, 88) = 6.00, *p* = 0.016, partial *η²* = 0.07), as well as craving for fat (*F*(1, 88) = 4.87, *p* = 0.030, partial *η²* = 0.05) and sweets (*F*(1, 88) = 5.91, *p* = 0.017, partial *η*² = 0.07).

Additionally, a correlation between age and FC was detected. In both patients with BD and HC, individuals who reported higher frequencies of fat craving tended to be older (patients: *r* = 0.21, *p* = 0.015; controls: *r* = 0.31, *p* = 0.003), while those with frequent craving for fast food tended to be younger (patients: *r* = −0.29, *p* < 0.001; controls: *r* = −0.30, *p* = 0.004).

In BD, 44.9% of individuals were smokers (*M* = 2.39, *SD* = 0.67) and those reported significantly more craving for fat than non-smokers did (*M* = 2.16, *SD* = 0.68) after controlling for sex and age (*F*(1, 136) = 13.10, *p* ≤ 0.001, partial *η²* = 0.09).

When controlling for age, craving for fast food was significantly positively correlated with BDI (*r* = 0.26, *p* = 0.003) and HAMD (*r* = 0.19, *p* = 0.029) scores, respectively.

No difference in FC between patients with BD type 1 and BD type 2 was found.

### 3.3. Leptin and Ghrelin

Data for leptin and ghrelin were available from 144 participants. Leptin levels differed significantly between patients with BD and HC, however this difference did not withstand controlling for sex and BMI. Female individuals had higher serum leptin levels than male individuals in patients (F(1, 80) = 54.02, *p* < 0.001, partial η² = 0.38) as well as in controls (F(1, 49) = 23.48, *p* < 0.001, partial η² = 0.33), even if controlled for BMI. No group or sex differences were found for ghrelin serum levels. In BD, ghrelin levels were negatively correlated with BMI (r = −0.29, *p* = 0.006). No differences in leptin and ghrelin levels between individuals with BD taking different psychotropic medication were found.

In patients, we found a significant negative correlation between ghrelin serum levels and frequencies of total FC (r = −0.37, *p* ≤ 0.001), craving for fat (r = −0.28, *p* = 0.008), craving for sweets (r = −0.23, *p* = 0.028), craving for carbohydrates (r = −0.32, *p* = 0.002) and craving for fast food (r = −0.37, *p* ≤ 0.001), respectively. In HC we found a significant negative correlation between ghrelin levels and craving for fat (r = −0.28, *p* = 0.047). No correlations between leptin levels and FC were found in BD. However, in HC we found a significant correlation between leptin and fast food craving (r = 0.30, *p* = 0.033).

### 3.4. Specific Cravings

In order to identify participants who reported cravings for one particular subcategory, median-splits for all subscales of the FCI where performed. Individuals who scored above the sample median on one subscale but below the median on the other tree subscales were denoted specific cravers (White et al. 2002). Overall, 22.3% of patients and 30.1% of HC were specific cravers. HC (7.5%) reported significantly more specific craving for carbohydrates than patients (2.2%), *χ^2^* (1) = 3.89, *p* = 0.048. No differences regarding the three other subscales were found. In the control group, women (37.9%) reported more specific craving (*χ2* (1) = 4.48, *p* = 0.034) than men (17.1%). Female HC (12.1%) were also more likely to be specific carbohydrate cravers than male individuals (none; *χ2* (1) = 4.57, *p* = 0.033). No other sex differences in specific craving were found.

Additionally, we aimed at identifying individuals with high FC across all subcategories. Participants who scored above the sample median on all four subscales of the FCI were deemed super cravers. In BD, 14.4% of individuals were super-cravers, in controls 7.5%. Nevertheless, no significant sex or group differences in the number of super-cravers were found. In patients, no differences in clinical parameters (sex, age, BMI, depressive symptoms, medication intake) between super-cravers and those reporting less craving were found.

### 3.5. Psychotropic Medication

In BD, individuals taking second-generation antipsychotics with an especially high risk of weight gain (olanzapine, quetiapine) reported higher frequencies of craving for sweet food items (*M* = 2.76, *SD* = 0.83) than those without such types of medication (*M* = 2.42, *SD* = 0.82; *F*(1, 130) = 4.19, *p* = 0.043, partial *η²* = 0.03). In contrast, patients currently taking lithium reported less total FC (*M* = 2.26, *SD* = 0.48) compared to those without lithium therapy (M = 2.55, SD = 0.50; *F*(1, 136) = 4.67, *p* = 0.033, partial *η²* = 0.03). Likewise, individuals with BD currently taking valproate reported less total FC (*F*(1, 130) = 7.19, *p* = 0.008, partial *η²* = 0.05) and less craving for sweets (*F*(1, 130) = 6.72, *p* = 0.011, partial *η²* = 0.05) than those not taking this medication.

## 4. Discussion

The aim of this investigation was to further elucidate the relationship between FC, clinical parameters, medication intake and appetitive peptides in a well-characterized sample of individuals with BD and HC.

Individuals with BD reported higher frequencies of total FC as well as craving for fat and fast food than HC. Additionally, BD individuals with more frequent cravings for fast food exhibited more depressive symptoms. While FC was negatively correlated with ghrelin levels, no correlation with leptin was found in BD.

Smokers with BD reported significantly more craving for high fat foods than non-smokers. Age was significantly associated with FC independent of group. Individuals with BP taking olanzapine or quetiapine reported higher frequencies of craving for sweet food, while patients currently taking lithium reported less total FC compared to those without lithium therapy. Likewise, patients currently taking valproate reported less total FC and less craving for sweets than those not taking valproate.

It has previously been shown that FC is often related to stress and negative affective states [17,19,54,55,56]. In a sample of more than thousands healthy adults, FC were associated with solitude, boredom, annoyance and depression in women [17]. Likewise, in an investigation by Massey and Hill [57] episodes of FC were correlated with feelings of anxiety, irritability, tension and emotional vulnerability. Individuals with BD, even during a period of euthymia, might exhibit more stress and negative affective states than HC and might therefore be prone to experience FC more frequently. Additionally, this investigation demonstrated that individuals with BD with more frequent cravings for fast food tended to exhibit more depressive symptoms. Fast food is often highly palatable, highly processed and tends to be rich in carbohydrates, sodium and fat [58]. It has been proposed that these attributes generate a “refined food addiction” that is associated with higher frequency of craving [59]. It could be hypothesized that individuals experiencing emotional distress or depressive symptoms might also be more susceptible to the addictive properties. There are various other factors such as convenience, cost, advertising and its association with youth identity that contribute to increased fast food consumption especially among adolescents and young adults [58,60]. In line with this, in our investigations, younger individuals among both BD and HC reported more frequent cravings for fast food. In general, previous reports have suggested a negative correlation between FC and age in accordance with the decline in appetite and other motivated behaviors seen in the elderly [19].

Whether food possesses actual addictive properties is still considered to be controversial [61]. However, some authors have suggested that similarities in craving for food vs. addictive substances exist [62]. Additionally, it was previously shown that ghrelin not only stimulates appetite and food intake but may also intensify representations of food, i.e., individuals are able to envision their favorite dishes better, therefore increasing the motivation to eat [63]. We hypothesized that this may as well be associated with an increased craving for food and expected to see a positive correlation between FC and ghrelin levels. Chao and colleagues demonstrated that higher levels of fasting ghrelin could predict future food carvings in a community sample [5]. Surprisingly though, in this investigation we found that in patients, ghrelin serum levels were inversely correlated to frequencies of total FC, craving for fat, craving for sweets, craving for carbohydrates, and craving for fast food, respectively. Interestingly, a recent study investigating the relationship between acylated ghrelin levels and the neural response to food stimuli in remitted major depressive disorder (MDD) via fMRI did find a positive association between ghrelin and the blood-oxygen-level-dependent (BOLD) response to high-calorie food in certain brain regions only in hyperphagic MDD. In hypophagic MDD a negative correlation between ghrelin and BOLD activity was found [64]. Although in this investigation individuals with BD were mostly euthymic, subclinical depressive symptoms including hypophagia might play a role in the inverse association observed.

This investigation did not find any correlations between levels of leptin and FC. This is consistent with an investigation by Chao et al. who failed to detect a relationship between leptin and the craving for food in a community sample [5]. In contrast to this, Macedo and Diez-Garcia demonstrated higher basal leptin levels in women with sweet cravings [65]. Furthermore, in previous work, leptin has been positively and negatively correlated with cravings for cocaine, nicotine and alcohol [66,67,68]. While these findings support the concept that the effects of leptin extend beyond energy homeostasis and might involve mechanisms underlying the search for rewarding substance, the heterogeneity of results warrants further investigations with well-defined population.

In our cohort, bipolar smokers exhibited higher FC compared to non-smoking patients. This goes in line with the results of Pepino et al. [10] who assessed FC in a cohort of 229 smoking and non-smoking women who were generally healthy but in part reported depressive symptoms. It has been previously shown that smokers consume more fat than non-smokers do [69,70] which might also be associated with more frequent cravings for fat. In the investigation at hand, individuals with BD where more likely to smoke than HC and associations between smoking and craving where only present in this subgroup of individuals with BD. Since symptoms of nicotine withdrawal such as irritability, anxiety and depression occur within 2–12 h after smoking the last cigarette [71], smokers might experience periods of stress brought on by withdrawal on a daily basis and might prefer to consume fatty—and starchy—foods to alleviate these symptoms [72]. In line with this, it is noteworthy that decreased serotonergic and dopaminergic function is associated with nicotine withdrawal [73]. Since neurotransmission is assumed to be altered [74,75] in BD, individuals with BD might be biologically prone to withdrawal symptoms and, consequentially, higher frequencies of craving for fat. On a behavioral level, individuals with BD might not tolerate states of irritability or anxiety to the same extend as HC. Additionally, craving for cigarettes may simply be confused with craving for foods by those who smoke [76].

In BD, individuals taking second-generation antipsychotics with an especially high risk of weight gain (olanzapine, quetiapine) reported higher frequencies of craving for sweet food items than those without such types of medication. The association between the craving for and subsequent consumption of carbohydrates and obesity, negative affective states as well as intake of psychotropic medication has been widely discussed. It has, however, been found that carbohydrate foods that are primarily craved, are, in fact mainly sweet, with sugar being the predominant carbohydrate as well as containing high amounts of fat [77]. The original definition of carbohydrate craving referred to a ‘ravenous appetite’ for chocolate, pastry and ice cream as a side effect of the tricyclic antidepressant amitriptyline [78], our findings might just be a manifestation of the previously described phenomenon. In contrast to this and somewhat surprising, patients currently taking lithium reported less total FC compared to those without lithium therapy. Likewise, individuals with BD currently taking valproate reported less total FC and less craving for sweets than those not taking valproate. Importantly, individuals with BD taking valproate reported fewer depressive symptoms in our cohort, which might have been a confounding factor.

### Limitations

Due to the cross-sectional design of this investigation, interpretations about the direction of the associations found cannot be drawn. Additionally, several factors pertinent to eating behavior, FC and appetite-related hormones such as sleep, dieting behavior, thyroid function or assessment of the menstrual cycle in woman (including premenstrual dysphoric disorder) have not been considered in this analysis. There is a strong correlation between obesity and binge eating disorder that is also relevant in BD [79]. It is of note here that none of the participants had a diagnosis of an eating disorder, however, this was not assessed formally in this investigation. Since we did only measure self-reported FC, we cannot draw conclusions about potential consequences in the form of actual consumption of food. However, multiple studies point to an association between FC and increased eating [80,81,82,83].

## 5. Conclusions

This investigation demonstrated differences in FC between individuals with BD and a healthy control group. Furthermore, associations between FC and psychotropic medication intake as well as smoking were shown. The underlying causes of obesity are multifaceted and include genetic, epigenetic and behavioral factors. However, FC might play a part in the increased prevalence of obesity in BD especially when the influence of medication and smoking behavior are taken into consideration. Across previous literature, the focus of analyses seems to have been the craving for carbohydrates and sweets and FC has been depicted as a predominately female phenomenon. This investigation has shown that cravings for savory, high fat foods, including fast food, might be of clinical relevance, that FC is significant in both female and male individuals, and that men might be, for once, the understudied gender.

The results presented are ambiguous and warrant further investigations. Obesity as well as mood disorders including bipolar depression will continue to present a medical and socioeconomic challenge with either few existing pharmacological interventions (in the case of obesity) or medication that is likely to be associated with multiple side effects, among them weight gain (in the case of affective disorders). Further explorations of appetite-related hormones as well as behavioral concepts like FC might not only facilitate psychopharmacological innovation, but might be the starting point in a multimodal therapeutic approach.

## Figures and Tables

**Table 1:** Differences in demographic and clinical characteristics between individuals with BD and HC stratified by sex.

	BD (*n* = 139)			HC (*n* = 93)			Statistics
	Male (*n* = 74)	Female (*n* = 65)	Statistics	Male (*n* = 35)	Female (*n* = 58)	Statistics	5.45 *
Age [years] (M, SD)	46.5 (14.1)	43.9 (13.7)	1.11	39.4 (16.1)	38.3 (15.7)	0.31	3.26 **
BMI [kg/m^2^] (M, SD)	29.4 (6.6)	26.8 (5.8)	2.43 *	26.0 (4.2)	23.7 (4.5)	2.44 *	4.93 ***
Smoking (%)	43.20	46.90	0.18	17.60	22.80	0.34	13.87 ***
Leptin [ng/mL] (M, Mdn, SD)	10.3; 6.8 (9.9) ^a^	19.9; 16.9 (13.5) ^a^	−3.89 ***	8.7; 5.4 (9.0) ^b^	12.9; 11.7 (7.5) ^c^	−1.82	2.23 *
							
Ghrelin [pg/mL] *(M, Mdn, SD)*	170.7; 104.3 ^a^ (265.0)	173.0; 156.8 ^a^ (127.0)	−0.05	162.0; 120.0 (121.0) ^d^	231.4; 177.9 (222.5) ^c^	−1.32	−0.87
BDI (M, SD)	13.9 (10.4)	15.0 (11.1)	−0.57	2.44 (2.76)	3.39 (3.41)	−1.37	11.48 ***
HAMD (M, SD)	5.5 (4.4)	5.8 (4.8)	−0.45	0.17 (0.48)	0.49 (1.35)	−1.32	12.70 ***
YMRS (M, SD)	1.3 (2.1)	0.7 (1.4)	2.20 *	0.25 (0.90)	0.22 (1.16)	0.12	3.73 ***
BD type I (%)	66.70	61.50	0.39	-	-	-	-
Illness duration [years] (M, SD)	18.74 (12.80)	20.27 (13.00)	−0.69	-	-	-	-
GAF (M, SD)	67.37 (13.58)	69.58 (12.96)	−0.95	-	-	-	-
Medication (n,%)							
Lithium	26 (34.2) ^e^	18 (27.7)	-	-	-	-	-
Anticonvulsives	21 (27.6) ^f^	19 (29.2) ^g^	-	-	-	-	-
Lamotrigine	6 (7.9) ^f^	10 (15.4) ^g^	-	-	-	-	-
Valproate	10 (13.2) ^f^	6 (9.2) ^g^	-	-	-	-	-
SGAs	41 (53.9) ^f^	42 (64.6) ^g^	-	-	-	-	-
Olanzapine	6 (7.9) ^f^	1 (1.5) ^g^	-	-	-	-	-
Quetiapine	21 (27.6) ^f^	26 (40) ^g^	-	-	-	-	-
FGAs	6 (7.9) ^f^	15 (23.1) ^g^	-	-	-	-	-
Antidepressants	49 (64.5) ^f^	42 (63.1) ^g^	-	-	-	-	-

Notes: ^a^ *n* = 46, ^b^ *n* = 21, ^c^ *n* =30, ^d^ *n* = 22, ^e^ *n* = 74, ^f^ *n* = 70, ^g^ *n* = 63. Abbreviations: Bipolar disorder (BD), healthy controls (HC), mean (M), standard deviation (SD), body mass index (BMI), Beck Depression Inventory (BDI), Hamilton Rating Scale for Depression (HAMD), Young Mania Rating Scale (YMRS), Global Assessment of Functioning (GAF), second generation antipsychotics (SGAs), first generation antipsychotics (FGAs). Significant statistic results from Chi-Square tests/t-tests are presented in bold letters (* *p* < 0.05, ** *p* < 0.01, *** *p* < 0.001).

**Table 2:** Differences in craving for the 28 food items of the FCI between individuals with BD and HC stratified by sex (analyzed by MANOVA).

	BD (*n* = 139)			HC (*n* = 93)			F
Food Item (M, SD)	Male (*n* = 74)	Female (*n* = 65)	F	Male (*n* = 35)	Female (*n* = 58)	F	
Fat	2.44 (0.68)	2.10 (0.62)	11.94 **	2.07 (0.66)	1.76 (0.56)	6.04 *	
Fried chicken	2.42 (1.01)	2.05 (1.05)	4.54 *	1.89 (0.93)	1.79 (0.91)	0.22	8.50 **
Hotdogs	2.26 (1.01)	1.85 (0.89)	6.42 *	2.00 (0.94)	1.64 (0.81)	3.86	3.44
Pork roast	2.45 (1.08)	1.74 (0.89)	17.60 ***	1.83 (0.86)	1.55 (0.78)	2.57	10.16 **
Fish fingers	2.20 (1.02)	2.06 (1.14)	0.59	1.77 (1.00)	1.64 (0.89)	0.45	9.32 **
Bacon	2.20 (1.10)	1.80 (0.97)	5.18 *	1.89 (1.08)	1.53 (0.84)	3.10	4.53 *
“Wiener Schnitzel”	2.82 (1.04)	2.40 (1.04)	5.76 *	2.26 (1.12)	2.03 (1.03)	1.00	10.58 **
Cold cuts	2.81 (1.24)	2.54 (1.20)	1.73	2.40 (1.27)	2.02 (1.05)	2.48	8.26 **
Steak	2.38 (1.07)	2.05 (1.15)	3.11	2.54 (1.25)	1.90 (1.003)	7.54 **	0.00
Sweet	2.50 (0.77)	2.63 (0.89)	0.90	2.23 (0.86)	2.40 (0.66)	1.22	
Cookies	2.70 (1.32)	2.83 (1.23)	0.35	2.26 (1.17)	2.71 (1.06)	3.63	2.95
Wafers	2.69 (2.51)	2.51 (1.34)	0.63	2.26 (1.22)	2.21 (1.17)	0.04	4.36 *
Candy	1.78 (1.02)	2.12 (1.13)	3.46	1.83 (1.04)	1.86 (1.22)	0.02	0.51
Chocolate	3.19 (1.16)	3.35 (1.34)	0.61	2.89 (1.23)	3.22 (1.23)	1.65	1.64
Tortes	2.27 (1.04)	2.46 (1.13)	1.08	2.06 (1.21)	2.07 (1.06)	0.02	4.08 *
Cake	2.53 (1.00)	2.60 (1.20)	0.15	2.06 (1.14)	2.66 (0.93)	7.64 **	2.04
Sweet pastries	2.35 (1.03)	2.52 (1.23)	0.81	2.09 (1.10)	2.36 (1.06)	1.46	2.00
Ice cream	2.46 (1.08)	2.65 (1.18)	0.95	2.40 (1.12)	2.14 (1.03)	1.32	3.56
Carbohydrates	2.36 (0.49)	2.36 (0.62)	0.00	2.14 (0.72)	2.18 (0.64)	0.10	
Rolls	2.58 (1.01)	2.46 (1.21)	0.40	2.29 (1.25)	2.21 (1.20)	0.10	3.05
Pancakes	2.23 (1.07)	2.08 (1.02)	0.74	1.94 (1.21)	1.83 (1.03)	0.24	3.38
Sponge cake	1.80 (0.90)	1.74 (0.89)	0.15	1.34 (0.84)	1.69 (0.92)	3.30	4.27 *
Toast	2.07 (1.01)	2.00 (0.98)	0.16	1.94 (1.11)	1.76 (0.84)	0.82	1.87
Rice	2.84 (0.92)	2.71 (1.23)	0.50	2.46 (1.17)	2.48 (1.30)	0.01	3.70
Baked potatoes	2.54 (1.04)	2.60 (1.14)	0.10	2.26 (1.17)	2.48 (1.17)	0.81	1.71
Pasta	3.04 (1.00)	3.08 (1.20)	0.04	2.80 (1.16)	2.95 (1.08)	0.39	1.52
Cereal	1.76 (0.95)	2.20 (1.33)	5.23 *	2.06 (1.16)	2.05 (1.30)	0.00	0.22
Fast food	2.40 (0.73)	2.20 (0.80)	2.33	2.18 (0.73)	2.10 (0.86)	0.39	
Hamburgers	2.35 (1.13)	2.05 (1.15)	2.49	2.17 (1.07)	1.84 (0.97)	2.29	1.64
French fries	2.24 (1.02)	2.11 (1.06)	0.59	2.03 (0.82)	1.74 (0.79)	2.82	5.00 *
Potato chips	2.22 (1.05)	2.14 (1.24)	0.16	2.00 (1.19)	1.90 (0.93)	0.22	2.32
Pizza	2.80 (0.90)	2.52 (1.02)	2.87	2.51 (1.01)	2.45 (0.96)	0.10	1.85

Abbreviations: Food craving inventory (FCI), Bipolar disorder (BD), healthy controls (HC), multivariate analysis of variance (MANOVA), mean (M), standard deviation (SD), bipolar disorder (BD), healthy controls (HC). Significant statistic results from t-tests are presented in bold letters (* *p* < 0.05, ** *p* < 0.01, *** *p* < 0.001).

## Data Availability

The data presented in this study are available on request from the corresponding author.
